# Associations between acquired antimicrobial resistance genes in the upper respiratory tract and livestock farm exposures: a case–control study in COPD and non-COPD individuals

**DOI:** 10.1093/jac/dkae335

**Published:** 2024-09-24

**Authors:** Beatrice Cornu Hewitt, Alex Bossers, Warner van Kersen, Myrna M T de Rooij, Lidwien A M Smit

**Affiliations:** Institute for Risk Assessment Sciences (IRAS), Utrecht University, P.O. Box 80178, Utrecht 3508 TD, The Netherlands; Institute for Risk Assessment Sciences (IRAS), Utrecht University, P.O. Box 80178, Utrecht 3508 TD, The Netherlands; Institute for Risk Assessment Sciences (IRAS), Utrecht University, P.O. Box 80178, Utrecht 3508 TD, The Netherlands; Institute for Risk Assessment Sciences (IRAS), Utrecht University, P.O. Box 80178, Utrecht 3508 TD, The Netherlands; Institute for Risk Assessment Sciences (IRAS), Utrecht University, P.O. Box 80178, Utrecht 3508 TD, The Netherlands

## Abstract

**Background:**

Livestock-related emissions have been associated with aggravations of respiratory symptoms in patients with chronic obstructive pulmonary disease (COPD), potentially by altering the respiratory resistome.

**Objectives:**

This study investigates the structure of the acquired oropharyngeal (OP) resistome of patients with COPD and controls, its interplay with the respiratory microbiome and associations with residential livestock exposure.

**Methods:**

In a matched case–control study in the rural Netherlands, we analysed OP swabs from 35 patients with COPD and 34 controls, none of whom had used antibiotics in the preceding 4 weeks. Resistome profiling was performed using ResCap, complemented by prior characterization of the microbiome via 16S rRNA-based sequencing. Residential livestock farm exposure was defined using distance-based variables alongside modelled concentrations of livestock-emitted microbial pollutants. We compared resistome profiles between patients with COPD and controls, examining alpha and beta diversity as well as differential abundance. Additionally, we assessed the interplay between the resistome and microbiome using co-occurrence networks and Procrustes analysis. Variations in resistome profiles were also analysed based on residential livestock exposures.

**Results:**

Patients with COPD exhibited higher resistome diversity than controls (Shannon diversity, *P *= 0.047), though resistome composition remained similar between groups (PERMANOVA, *P *= 0.19). Significant correlations were observed between the OP resistome and microbiome compositions, with distinct patterns in co-occurrence networks. Residential exposure to livestock farms was not associated with resistome alterations.

**Conclusions:**

Our findings reveal the COPD airway as a hospitable environment for antimicrobial resistance genes, irrespective of recent antimicrobial usage. Demonstrating the interplay between the resistome and microbiome, our study underscores the importance of a deeper understanding of the resistome in respiratory health.

## Introduction

The impact of livestock farm emissions on the respiratory health of neighbouring residents is an important concern within public health. Previous studies have established significant associations between exposure to these emissions and respiratory morbidity and mortality.^1–8^ Livestock farm emissions constitute a complex mixture of gases and particulate matter,^9–11^ comprising microbiological components such as endotoxins and antimicrobial resistant (AMR) bacteria,^12–16^ thereby presenting a multifaceted challenge. Notably, it has been observed that patients with chronic obstructive pulmonary disease (COPD) residing in close proximity to farms demonstrate increased risks of airway inflammation, cough and dyspnoea, compared with those residing further away.^17,18^

In a recent study, researchers discovered an association between residential exposure to livestock-related air pollution and increased microbial richness in the upper respiratory tract, highlighting the potential influence of the livestock environment on the microbiome.^19^ The composition of the airway microbiome plays a significant role in the onset and progression of lung diseases. Previous research consistently demonstrates that alterations in the airway microbiome are associated with the development and progression of COPD.^20–22^ These studies indicate a decline in microbial diversity as COPD progresses and during acute exacerbations.^23–27^ The microbiome and resistome (the complete collection of acquired bacterial genes potentially responsible for acquired antimicrobial resistance) reciprocally interact.^28,29^ The microbiome provides a diverse ecosystem that facilitates the acquisition and preservation of antimicrobial resistance genes (ARGs) through gene transfer. Simultaneously, the resistome influences the microbiome by conferring advantages to microorganisms under selective pressures, influencing their abundance and composition based on their resistance potential.

Despite the growing interest in microbiome research, the investigation of the relationship between the respiratory resistome and respiratory health remains limited. Although studies have demonstrated the transmission of AMR bacteria in occupational settings to farmworkers from farm dust,^30–32^ the impact of residential exposure to livestock emissions on the respiratory resistome remains unexplored. Gaining insight into the resistome among both patients with COPD and control individuals allows for the identification of disease-specific resistome patterns and enhances our understanding of the impacts of livestock farm exposure. Investigating the interplay between the microbiome and resistome unravels intricate dynamics, potentially providing valuable insights into interactions of ARGs within the airways. Given the escalating global health threat posed by AMR,^33^ and the potential of ARGs within the airway to lead to severe and difficult-to-treat infections, it is important to address this critical knowledge gap by shedding light on the structure of the respiratory tract resistome in health and disease and by investigating the dynamics between residential exposure and the resistome.^33^

This study characterized the acquired ARGs in the oropharynx of rural residents living in a livestock-dense region. We aimed to investigate associations between the oropharyngeal (OP) resistome composition and (i) COPD status, (ii) the OP microbiome composition and (iii) residential exposure to livestock-related microbial emissions. In this case–control study, we collected OP samples from 35 patients with COPD and 34 matched controls. To characterize the resistome, we employed resistome-enrichment (ResCap) metagenomic shotgun sequencing. We hypothesize that the OP resistome of patients with COPD is inherently different to that of the controls and that the microbiome and resistome mutually shape one another. Furthermore, we anticipate that residential exposure to livestock-related emissions will induce alterations in the load and structure of the OP resistome, potentially contributing to the aggravation of respiratory symptoms in patients with COPD.

## Methods

### Study design and population

**Figure 1.**
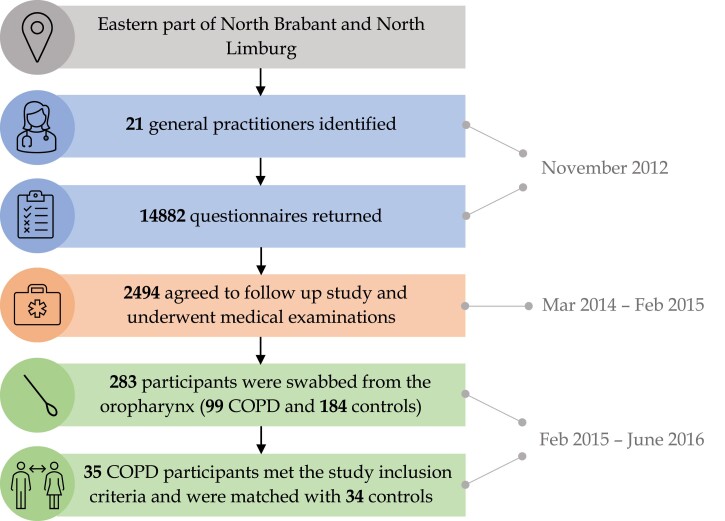
A flow chart of the participant selection procedure from the VGO study, illustrating the procedure for the selection of matched patients with COPD and controls. This figure appears in colour in the online version of *JAC* and in black and white in the print version of *JAC*.

The study population comprises rural residents in the Netherlands, both with and without a diagnosis of COPD. This study is nested within the VGO programme (Dutch acronym for ‘livestock farming and the health of neighbouring residents’) initiated in 2012 to investigate the health effects associated with residential proximity to livestock farms. The initial selection procedure for VGO participants was previously outlined.^18,34^ Subsequently, participants were selected for a COPD case–control microbiome study,^19^ from which we selected participants for this resistome study. The inclusion criteria stipulated that patients with COPD must (i) live in the VGO area in 2015–16, (ii) have a COPD diagnosis based on spirometry results and a categorization into Global Initiative for Chronic Obstructive Lung Disease (GOLD) Stages 1–3 and (iii) be aged ≥ 40 years. The exclusion criteria included: (i) any participants with missing data, (ii) farm workers, (iii) residing on a farm, (iv) current smokers and (v) recent use (within the past 4 weeks) of oral antibiotics (Figure 1). Control participants were carefully matched with the patients with COPD based on age category, sex, smoking status (never, ex-smoker) and farm childhood.

### Sample collection

OP samples from 35 patients with COPD and 34 control participants were used for this study, collected during home visits in the period February 2015 to July 2016 in addition to three field blanks. Previous research affirms the resemblance between bacterial communities in the oropharynx and lungs, establishing OP samples as a valuable proxy for our research.^35^ Copan eSwabs were used for sampling, and these were stored in 1 mL liquid Amies Medium (483CE, Copan Diagnostics Inc., CA, USA). During transportation, these samples were stored on ice until transfer to the −80°C freezer on the same day. Blanks were unused swabs treated on-site using the same procedure as participant samples. The DNA extracts were identical to those described in the microbiome analysis protocol outlined by van Kersen *et al*..^19^

### Sequencing and bioinformatics

Resistome characterization of the OP samples was performed using the ResCap method, a shotgun metagenomic enrichment approach tailored for resistome analyses.^36^ The ResCap procedure consists of four main steps: (i) total microbial DNA isolation,^19^ (ii) whole-metagenome shotgun library construction (Roche SeqCap EZ workflow), (iii) hybridization and enrichment and (iv) enriched library deep sequencing (Illumina), as previously described.^36^ After quality control and trimming of the raw sequences, these were mapped against the ResFinder database of acquired ARGs.^37^ Mapping data were subsequently normalized for ARG length and microbial load using 16S rRNA qPCR. For additional information on sequencing details and bioinformatics, refer to Text S1.

### Residential livestock exposure assessment

Residential exposure for each participant was assessed using their geocoded home address. To investigate potential factors influencing the structure of the OP resistome, we considered both proxies of livestock exposure and predicted concentrations of livestock-related emissions derived from previously established models.^38,39^ Exposure proxies were computed using Geographic Information System (GIS) software (ArcGIS; version 10.2.2, Esri)^40^ using geolocated information on livestock farms alongside geocoded residential addresses, as previously described.^13^ Proxies included general, species-specific and farm type-specific variables. Land-use regression, dispersion and random forest models were applied to estimate residential exposure to generic and more specific livestock-related emissions. These emissions included PM_10_, endotoxin, two livestock commensals (*Escherichia coli* and *Staphylococcus* spp.) and two ARGs (*tetW* and *mecA*); all frequently encountered on livestock farms and in their surrounding areas.^16,41–43^ All models were previously developed and validated for our specific geographic region, and they were employed in our study to predict the annual average residential exposure to these emissions.^38,39^

### Data analysis

All statistical analyses were conducted using R version 4.2.2 (2022-10-31).^44^ The OP resistome was characterized using established methods, including evaluations of within-sample diversity (alpha diversity), between-sample compositional differences (beta diversity) and in-depth investigations of differences in ARG abundances [differential abundance (DA) analysis]. Firstly, we compared these resistome characteristics between patients with COPD and controls. Secondly, we examined the relationship between the microbiome and resistome using Procrustes analysis and co-occurrence networks. Finally, we examined the potential association between the OP resistome and residential livestock exposure. For a comprehensive explanation of the applied statistical methods, refer to Text S2.

## Results

### Study population

Characteristics of the study population, stratified by COPD status, are presented in Table 1. The majority of participants were male (59%), with a mean age of 61 years. Among patients with COPD, all were categorized into GOLD Stage 1 or 2 (51% and 49%, respectively), indicating mild and moderate COPD according to the GOLD criteria. For detailed livestock exposure proxy values for each participant, refer to Table S2 (available as Supplementary data at *JAC* Online).

**Table 1.** Baseline characteristics of patients with COPD and control participants included in the study

	Patients with COPD	Controls	*P* value
	(*n* = 35)	(*n* = 34)	
**Gender**			
Male	21 (60.0%)	20 (58.8%)	1
Female	14 (40.0%)	14 (41.2%)	
**Age**			
Mean (SD)	61.5 (7.36)	60.2 (7.82)	0.473
Median (min, max)	62.6 (43.9, 71.6)	62.7 (41.8, 70.6)	
**BMI**			
Mean (SD)	26.7 (4.15)	27.5 (4.40)	0.473
Median (min, max)	25.9 (20.1, 34.1)	26.3 (21.4, 39.2)	
**COPD GOLD grade**			
0	0 (0%)	34 (100%)	<0.001
1	18 (51.4%)	0 (0%)	
2	17 (48.6%)	0 (0%)	
**Childhood on farm**			
No	22 (62.9%)	22 (64.7%)	1
Yes	13 (37.1%)	12 (35.3%)	
**Smoking status**			
Former smoker	27 (77.1%)	26 (76.5%)	1
Never	8 (22.9%)	8 (23.5%)	
**Pack-years of cigarettes smoked**			
Mean (SD)	16.8 (15.1)	13.8 (22.0)	0.506
Median (min, max)	16.7 (0, 54.6)	7.25 (0, 117)	
**Education level**			
No	22 (62.9%)	22 (64.7%)	1
Yes	13 (37.1%)	12 (35.3%)	
**Number of farms within 3 km radius**			
Mean (SD)	82.7 (30.1)	90.0 (23.1)	0.265
Median (min, max)	78.0 (13.0, 137)	89.5 (20.0, 129)	

For continuous variables, data are presented as mean (SD) and median (min, max), and *P* values were derived from *t* tests between COPD and control groups. For categorical variables, data are presented as number (%) per category and *P* values were derived from the χ^2^ test of independence.

### Oropharyngeal resistome of patients with COPD and controls

**Figure 2.**
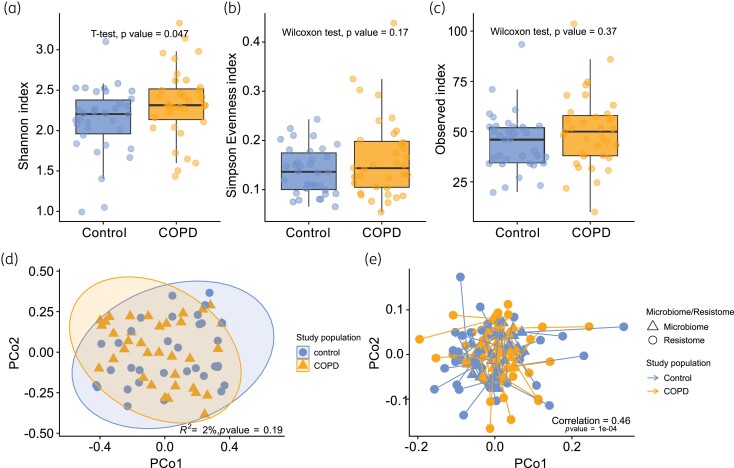
Comparison of the OP resistomes of patients with COPD and controls. Alpha diversity indices (a) Shannon, (b) Simpson’s evenness and (c) observed richness for COPD and control OP resistome samples. *P* values were derived from *t* tests and Wilcoxon’s rank sum tests. (d) PCoA plot based on the Bray–Curtis dissimilarity matrix of the OP resistome of patients with COPD and controls. Ellipses show the 95% CI for the centroid of each group. Points represent the resistance gene communities of one participant. The first two principal coordinates of the PCoA (Axis 1 and Axis 2) explained 28.5% and 17.2% of the variance, respectively. (e) Procrustes superimposition plot showing the association between microbiome and resistome PCoAs. The *protest* function was used to calculate the correlation coefficient and *P* value for paired participant samples using 9999 permutations. This figure appears in colour in the online version of *JAC* and in black and white in the print version of *JAC*.

The COPD group exhibited significantly higher Shannon diversity compared with the control group (two-sample *t* test; *P* value = 0.047) (Figure 2a). Observed richness and Simpson’s evenness did not reveal significant differences between patients with COPD and controls (Figure 2b and c).

**Figure 3.**
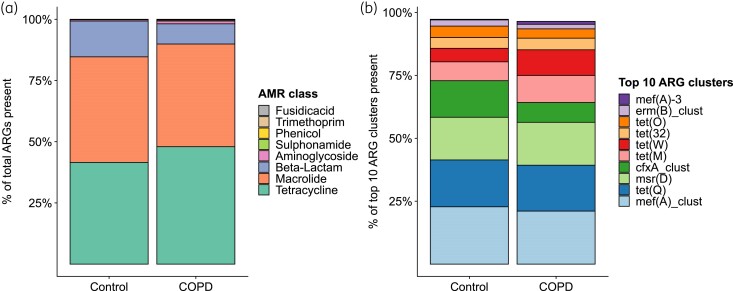
Relative resistome abundances across patients with COPD and controls where (a) shows the relative abundances ARGs grouped by their AMR class and (b) shows the relative abundance of the top 10 ARGs (90% gene identity) within the total resistome. ARG counts have been (relatively) rarefied, gene length-corrected and 16S qPCR-corrected prior to scaling to 100%. This figure appears in colour in the online version of *JAC* and in black and white in the print version of *JAC*.

Across the entire study population, we identified 85 distinct ARGs (90% identity clusters). Figure 3(a) displays the relative abundances of ARGs, grouped by their AMR class, while Figure 3(b) presents the relative abundances of the top 10 ARGs, which collectively explain 99.2% of the data from the control population and 96.4% from the COPD population. Within the top 10 ARGs across all samples, 50% conferred tetracycline resistance, 40% macrolide resistance and 10% beta-lactam resistance. The predominant ARG observed in all samples was *mef(A)_clust*, associated with macrolide resistance. Relative abundance patterns of the eight AMR classes did not appear to differ considerably between COPD and control groups.

In each group, we identified eight core ARGs (see Text S2 for definition), conferring resistance potential to tetracyclines, macrolides and beta-lactams. Our analysis revealed an identical core resistome shared between patients with COPD and controls (Figure S1). Notably, the two ARGs that were found in low quantities in the blank samples exhibited no overlap with the resistome of patients with COPD and controls. A heatmap showing the abundance of ARGs across the groups is shown in Figure S2. No distinct clustering patterns were observed between patients with COPD and controls, as depicted in the principal coordinates analyses (PCoAs) (PERMANOVA; *P* value = 0.19, *R*^2^ = 0.0201) (Figure 2d). Similarly, DA analysis using DESeq and ALDEx did not reveal any statistically significant differentially abundant ARGs associated with COPD status, as detailed in Tables S3 and S4.

### Association between the oropharyngeal resistome and microbiome

Procrustes analysis demonstrated a moderate yet significant correlation between PCoA ordinations of the resistome and microbiome from the same individuals (correlation = 0.46, *P* value* *< 0.001), which was absent when microbiome participant IDs were randomized (correlation = 0.16, *P* value* *= 0.32). The paired Procrustes superimposition plot is depicted in Figure 2(e).

**Figure 4.**
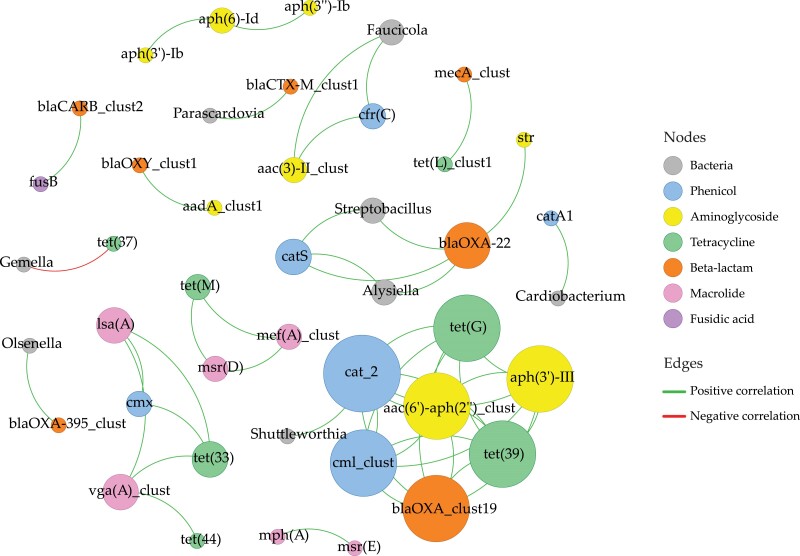
Bacteria–ARG and ARG–ARG co-occurrence network, based on correlation analysis. Nodes representing bacteria are named on their genus level, and nodes representing ARGs are named by their 90% cluster identity name. Nodes representing bacteria are coloured grey, and the colour of nodes representing ARGs shows their AMR class. Node size is proportional to the nodal degree (number of connections in the network). Edges show strong (Spearman’s |ρ| ≥ 0.6) and significant [*P* value (BH-adjusted) < 0.01] pairwise correlations. Edges coloured in green represent the positive correlations, while edges in red represent the negative correlations. This figure appears in colour in the online version of *JAC* and in black and white in the print version of *JAC*.

Co-occurrence correlation analysis between bacteria and ARGs uncovered associations among various ARGs and bacterial genera, as well as interactions among the ARGs themselves (Table S5a and b). Seven bacterial genera exhibited positive associations with one or more specific ARGs. Figure 4 illustrates a co-occurrence network depicting these correlations, with nodes representing bacteria and ARGs and edges representing positive and negative correlations. Notably, *cat_2* ARG (conferring phenicol resistance) emerged as central in the network, exhibiting the highest degree centrality (number of connections). Co-occurrence patterns were observed within the same AMR class and across different AMR classes, without significant negative correlations among ARGs. Bacteria–ARG correlations revealed a smaller cluster involving *Streptobacillus*, *Alysiella* and ARGs *cat_2* and *blaOXA-22* (phenicol and beta-lactam resistance genes). *Gemella* exhibited a significant negative correlation with *tet(37)* (a tetracycline ARG), representing the sole negative correlation identified.

### Association between the oropharyngeal resistome and livestock exposure

Livestock exposure analysis revealed no significant differences in resistome composition and diversity across individuals with differing levels of exposure, regardless of the specific exposure variable used. Detailed results are given in Tables S6 (PERMANOVA), S7 and S8 (analysis of alpha diversity indices). DA analyses, exploring potential relationships between residential livestock exposure variables and ARG expression, also did not reveal any significant differences (both DESeq and ALDEx methods produced Benjamini–Hochberg (BH)-corrected *P* values > 0.05).

## Discussion

The primary aim of our study was to advance our understanding of the respiratory resistome in rural residents, both with and without COPD. Alongside this, we aimed to conduct a comparative analysis of the resistome and microbiome compositions among the study participants. Additionally, we sought to examine the potential associations between the respiratory resistome and residential exposure to microbial emissions from livestock farms.

### The oropharyngeal resistome of patients with COPD versus controls

Consistent with our hypothesis, individuals with COPD demonstrated higher resistome alpha diversity compared with controls, even in the absence of antibiotic usage within the 4 weeks preceding sampling. Compelling evidence suggests that recurrent antibiotic use in individuals with COPD contributes significantly to the increased occurrence of phenotypic antibiotic resistance.^45^ On average, individuals with COPD tend to use antimicrobial agents more frequently, exerting a selection pressure for ARG expression. Additionally, chronic inflammation within the COPD airway can lead to the production of excess exudate which creates conditions that are more favourable for the survival and growth of microorganisms, including AMR bacteria.^46^ Consequently, individuals with altered immune function, such as those with COPD, may exhibit higher levels of AMR bacteria, even without increased antimicrobial usage, thus possibly leading to a higher diversity of ARGs. Despite this elevated resistome diversity in patients with COPD compared with controls, no differences in composition or overall resistome load [Fragments Per Kilobase of transcript per Million mapped reads (FPKM)] were observed between the two groups.

### Associations between the oropharyngeal resistome and microbiome

Having both resistome and microbiome data for our study participants, we had the unique opportunity to investigate their relationship. A strong correlation was observed between the compositional differences in the resistome and microbiome, suggesting a degree of interdependency. The resistome composition is likely influenced by the presence of specific bacterial species within the microbiome that can be reservoirs for ARGs. Additionally, host-specific factors, such as genetics, immune responses and exposure history, may contribute to the observed correlations. Despite significant overlap between the two compositions shown in the Procrustes analysis, resistome alpha diversity did not correlate with microbial alpha diversity.

Co-occurrence network analysis offered more nuanced insights into the complex relationships among ARGs and bacteria and between ARGs themselves. The distinct clusters of ARGs, which were not directly associated with a specific bacterium, suggest shared genetic elements or co-localization on mobile genetic elements. Positive associations between specific ARGs and bacterial genera imply multi-drug resistance or functional relationships, potentially identifying these bacteria as ARG reservoirs. Alternatively, associations may indicate shared ecological niches. The negative correlation between *Gemella* and *tet(37)* suggests that the presence of *Gemella* may exert an influence on *tet(37)*, or the possibility that another bacterium carrying *tet(37)* might be more prevalent or involved in competitive interactions with *Gemella*, thus unveiling intricate and dynamic interactions within this airway microbial ecosystem.

### Livestock exposure and its impact on the oropharyngeal resistome

Examining the relationship with livestock exposure, our analyses did not reveal any discernible shifts in resistome composition, relative abundance of ARGs, or alpha diversity associated with livestock exposure. The persistent presence of ARGs in the airways suggests a consistent underlying resistome across all study participants. It is crucial to acknowledge that all participants in our study had some degree of livestock exposure, given the overall high livestock density in the study region, thereby limiting the contrast between exposure levels, and possibly hindering our ability to discern potential differences.

### Study strengths and limitations

Strengths of our study include the use of novel ARG-enriched shotgun metagenomics to characterize the OP resistome.^36,47^ This approach offers a comprehensive view of the resistome along with their relative abundances which surpasses the limitations of conventional methods such as antibiotic susceptibility testing or qPCR, as previously demonstrated.^48^ Secondly, we assessed exposure to livestock-related emissions through a multifaceted approach, incorporating distance-based variables and predicted microbial exposures based on models developed and validated for our study region.^39^ Furthermore, the OP microbiota composition of the same study population was previously characterized utilizing 16S rRNA-based sequencing.^19^ This multi-omics approach enabled us to decipher potential associations between paired microbiome and resistome samples, revealing co-occurrence patterns between specific ARGs and bacteria.

Our study has certain limitations, primarily stemming from its exploratory nature. Notably, the generalizability of our findings to broader populations may be constrained by the homogeneity of the participants as they are all selected from the southern part of the Netherlands with relatively high livestock exposure levels. Additionally, the modest sample size likely compromised our ability to detect significant differences or associations where they may be present. It is also important to note that our study assessed ARGs, offering insights into the antimicrobial potential of the identified genes rather than their actual phenotypic resistance. While ARGs often show a strong correlation with resistance phenotypes, it is crucial to acknowledge the potential for discrepancies.^49^ Furthermore, our study lacks information on home or work-related exposures other than residential exposure to livestock farming, which could significantly influence the resistome. Additionally, our research exclusively involved participants with mild to moderate COPD, potentially limiting the case–control contrast in our comparisons. Respiratory diseases like COPD are typically more closely associated with microbiota in the lower respiratory tract. However, for practical reasons, we utilized OP samples as a proxy, as studies have demonstrated that the microbiota of these samples bears a closer resemblance to the lung microbiota than their nasopharyngeal counterparts.^50^ However, it is essential to recognize that OP samples, collected from the upper respiratory tract, may not perfectly mirror the microbiota in the lower respiratory tract.

### Study implications

Our findings revealed the pervasive presence of ARGs in both individuals with and without COPD living in a livestock-dense region, raising attention to potential clinical implications. Future research is needed to assess the risks associated with the presence of ARGs in the airways, particularly in the context of respiratory infections and treatment options. Livestock exposure did not emerge as a modifier of the OP resistome composition, consistent with microbiome analysis results from a previous study where no compositional changes were observed.^19^ To our knowledge, only one prior study has investigated the upper airway resistome in relation to livestock exposure. Conducted in an occupational setting, the study compared OP resistomes of 23 farmworkers with those of 12 nearby villagers. The findings revealed heightened abundance of ARGs associated with farm exposure, influenced by specific farm-related tasks and their duration.^51^ Another study, specifically focussing on nasal methicillin-resistant *Staphylococcus aureus* carriage, revealed an increased prevalence associated with residential proximity to livestock farms.^52^

Comprehensively understanding the impact of different degrees and types of livestock exposure on the resistome and its clinical implications requires further investigation. We recommend for future research that studies encompass diverse geographic and demographic settings to enhance generalizability, including a broader range of exposure levels across participants. The widespread detection of airborne ARGs raises concerns about their contribution to the dissemination of AMR, warranting additional research to evaluate associated risks. While we examined livestock exposure and COPD status as contributing factors to AMR, comprehensive investigations into various host-specific factors are essential for a better understanding of their influence.

In conclusion, this study provides novel insights into the structure of the respiratory resistome and its interplay with the microbiome, residential exposure to livestock farm emissions and respiratory health. Developments in cutting-edge technologies such as metagenomic shotgun sequencing^36,47^ have enabled the elucidation of the structure of the resistome in OP samples of our rural study population and the investigation of its determinants. These findings make valuable contributions to the emerging field of respiratory microbiome and resistome research, highlighting the importance of comprehending the interplay between the resistome and microbiome in disease dynamics. The identified associations between exposure and respiratory symptoms in individuals with COPD emphasize the need for comprehensive studies that integrate environmental, microbiological and clinical perspectives to unravel these complex interactions. This ‘One Health’ approach aligns with global efforts to both mitigate AMR and enhance our understanding of respiratory diseases by recognizing the interconnectedness of human health, animal health and the environment.

## Supplementary Material

dkae335_Supplementary_Data

## Data Availability

Metagenomic sequencing data were deposited in the NCBI Sequence Read Archive (SRA) under BioProject accession number PRJNA1049329. The phyloseq object used for this project is available at Zenodo (https://zenodo.org/records/10104830). Scripts used for the analyses and figures are available at https://github.com/BeatriceCornuHewitt/VGO_COPDcaco_resistome.

## Supplementary data

Figures S1 and S2 and Tables S2, S3, S4, S6, S7 and S8 are available as Supplementary data at *JAC* Online.
