# Supplementary material for: Associations between acquired antimicrobial resistance genes in the upper respiratory tract and livestock farm exposures: a case–control study in COPD and non-COPD individuals
Source: J Antimicrob Chemother. 2024 Sep 24;79(12):3160–8. doi: 10.1093/jac/dkae335 (PMC11638102; doi:10.1093/jac/dkae335)
Supplement: dkae335_Supplementary_Data [file dkae335_supplementary_data.docx]

**Supporting Information**

# Title

Associations Between Acquired Antimicrobial Resistance Genes in the Upper Respiratory Tract and Livestock Farm Exposures: A Case-Control Study in COPD and Non-COPD individuals

**Running title**

Respiratory Resistome in COPD and Farm Exposure

# Authors

Beatrice CORNU HEWITT^1,^*, Alex BOSSERS^1^, Warner VAN KERSEN^1^, Myrna M.T. DE ROOIJ^1^, Lidwien A.M. SMIT^1^

^1^ Institute for Risk Assessment Sciences (IRAS), Utrecht University, Utrecht, The Netherlands

* Corresponding author. E-mail address: [b.cornuhewitt@uu.nl](mailto:b.cornuhewitt@uu.nl). Telephone: +31 (0)30 - 253 24 89. Address: Institute for Risk Assessment Sciences (IRAS), P.O. Box 80178, 3508 TD Utrecht, The Netherlands

# Author ORCiDs:

Beatrice CORNU HEWITT: <https://orcid.org/0000-0002-4594-4393>

Alex BOSSERS: <https://orcid.org/0000-0002-6586-717X>

Warner VAN KERSEN: <https://orcid.org/0000-0002-8873-0475>

Myrna M.T. DE ROOIJ: <https://orcid.org/0000-0002-6560-4839>

Lidwien A.M. SMIT: <https://orcid.org/0000-0003-0292-0946>

**Text S1 Methods – Sequencing and Bioinformatics**

Individually barcoded libraries from each sample were enriched in equimolar pools of eight and subsequently sequenced with the Illumina NovaSeq 6000 using 150 bp paired-end (PE) sequencing with 16 million PE clusters per enrichment pool (GenomeScan, Leiden, the Netherlands). Read pairs were mapped against the ResFinder database^1^ (accessed 27^th^ January 2020) using the BBMap global aligner algorithm.^2^ ResFinder gene counts underwent correction for relative library volume inputs to enhance the quantitative accuracy of the procedure. These corrections covered every normalisation step in the laboratory process, including the initial input DNA quantities, library construction, and final enrichment volumes, as previously detailed and validated in prior work (Table S1 provides the correction factors calculated per sample).^3^ Subsequently, ARG-mapped reads were normalised for their respective gene length (in base pairs), and sequencing depth for each sample (defined as the total number of bacterial counts in each sample as obtained from 16S rRNA qPCR^4^). The normalised ResFinder gene counts were expressed as fragments per kilobase ARG per million bacterial fragments (FPKM) (Formula S1).

**Formula S1.**  $ARG FPKM =\frac{{N ARG mapped reads}/{ARG length}}{N bacterial reads}*{10}^{9}$

In order to address potential challenges associated with ambiguous read pair mapping to closely related ARG sequences within the ResFinder database, which initially comprised only acquired ARGs, we agglomerated sequences at a broader level. Specifically, we aligned them using a 90% gene identity threshold, following a methodology consistent with previous studies. Initially, CD-HITest was employed to establish 90% identity clusters of ARGs. These clusters underwent subsequent refinement through manual curation, resulting in a taxonomy-like organisation.^3^ Additional metadata, taxonomic information and ARG FPKMs were subsequently integrated into a phyloseq object^6^ for further analysis.

**Table S1.** Individual and total correction factors applied per sample

| **ID** | **16S qPCR (ng/ml)** | **DNA clariostar (ng/µl)** | **DNA clariostar (µl)** | **DNA clariostar (ng)** | **Lib cycle** | **Enrich pool #** | **Lib conc (ng/µl)** | **Lib pool (µl)** | **Final lib pool (ng)** | **Total read pairs** | **ResFinder read pairs** | **On target %** | **Template correction** | **Lib correction** | **Total correction** | **Total Correction Maxed** | **Relative Correction read pair counts** |
| --- | --- | --- | --- | --- | --- | --- | --- | --- | --- | --- | --- | --- | --- | --- | --- | --- | --- |
| 10279 | 0.85336 | 1.019 | 9.8 | 10.0 | 1 | 5 | 26.402 | 4.7 | 125.00 | 2373597 | 379515 | 15.99 | 9.895 | 1.211 | 0.083421 | 0.096559 | 36646 |
| 10839 | 0.17450 | 0.968 | 10.3 | 10.0 | 1 | 1 | 26.515 | 4.7 | 125.00 | 2471584 | 634040 | 25.65 | 10.416 | 1.206 | 0.079585 | 0.092119 | 58407 |
| 11143 | 0.18290 | 1.633 | 6.1 | 10.0 | 1 | 1 | 25.811 | 4.8 | 125.00 | 1165689 | 55551 | 4.77 | 6.175 | 1.239 | 0.130694 | 0.151277 | 8404 |
| 11502 | 0.08720 | 0.821 | 12.2 | 10.0 | 1 | 1 | 25.640 | 4.9 | 125.00 | 1255207 | 107769 | 8.59 | 12.281 | 1.247 | 0.065272 | 0.075552 | 8142 |
| 11588 | 0.17250 | 1.053 | 9.5 | 10.0 | 1 | 1 | 25.796 | 4.8 | 125.00 | 2674467 | 452998 | 16.94 | 9.575 | 1.240 | 0.084226 | 0.097491 | 44163 |
| 11814 | 0.12690 | 0.249 | 35.0 | 8.7 | 1 | 2 | 20.622 | 6.1 | 125.00 | 887769 | 22281 | 2.51 | 35.291 | 1.551 | 0.018269 | 0.021147 | 471 |
| 12927 | 1.82460 | 3.190 | 3.1 | 10.0 | 1 | 1 | 25.357 | 4.9 | 125.00 | 1685158 | 145192 | 8.62 | 3.161 | 1.261 | 0.250814 | 0.290316 | 42152 |
| 12965 | 0.21290 | 0.601 | 16.6 | 10.0 | 1 | 1 | 25.700 | 4.9 | 125.00 | 1871727 | 110638 | 5.91 | 16.777 | 1.245 | 0.047893 | 0.055436 | 6133 |
| 12980 | 0.42870 | 1.790 | 5.6 | 10.0 | 1 | 1 | 25.462 | 4.9 | 125.00 | 1463530 | 71976 | 4.92 | 5.633 | 1.256 | 0.141322 | 0.163579 | 11774 |
| 13571 | 0.54420 | 2.639 | 3.8 | 10.0 | 1 | 1 | 25.744 | 4.9 | 125.00 | 3470256 | 148657 | 4.28 | 3.821 | 1.242 | 0.210659 | 0.243836 | 36248 |
| 13743 | 1.35410 | 1.655 | 6.0 | 10.0 | 1 | 2 | 26.220 | 4.8 | 125.00 | 3237126 | 209784 | 6.48 | 6.092 | 1.220 | 0.134553 | 0.155745 | 32673 |
| 13764 | 0.09770 | 2.138 | 4.7 | 10.0 | 1 | 2 | 25.402 | 4.9 | 125.00 | 582849 | 43021 | 7.38 | 4.716 | 1.259 | 0.168399 | 0.194921 | 8386 |
| 13908 | 1.17350 | 1.996 | 5.0 | 10.0 | 1 | 2 | 25.580 | 4.9 | 125.00 | 4893223 | 570010 | 11.65 | 5.052 | 1.250 | 0.158316 | 0.183250 | 104454 |
| 14587 | 0.73210 | 1.554 | 6.4 | 10.0 | 1 | 2 | 25.374 | 4.9 | 125.00 | 2631668 | 389086 | 14.78 | 6.488 | 1.261 | 0.122265 | 0.141521 | 55064 |
| 15035 | 0.91214 | 1.478 | 6.8 | 10.0 | 1 | 9 | 29.270 | 4.3 | 125.00 | 3544368 | 412592 | 11.64 | 6.822 | 1.093 | 0.134141 | 0.155267 | 64062 |
| 15391 | 0.32680 | 1.517 | 6.6 | 10.0 | 1 | 2 | 24.576 | 5.1 | 125.00 | 1153857 | 142975 | 12.39 | 6.647 | 1.301 | 0.115601 | 0.133807 | 19131 |
| 15446 | 1.08670 | 1.906 | 5.2 | 10.0 | 1 | 2 | 28.010 | 4.5 | 125.00 | 2939096 | 1168336 | 39.75 | 5.290 | 1.142 | 0.165539 | 0.191610 | 223865 |
| 15573 | 0.58631 | 1.122 | 8.9 | 10.0 | 1 | 2 | 31.156 | 4.0 | 125.00 | 2296405 | 193749 | 8.44 | 8.987 | 1.027 | 0.108392 | 0.125463 | 24308 |
| 16236 | 0.64130 | 1.358 | 7.4 | 10.0 | 1 | 3 | 26.128 | 4.8 | 125.00 | 1421730 | 185399 | 13.04 | 7.425 | 1.224 | 0.110020 | 0.127347 | 23610 |
| 16716 | 0.16820 | 0.454 | 22.0 | 10.0 | 1 | 3 | 25.402 | 4.9 | 125.00 | 948400 | 128850 | 13.59 | 22.209 | 1.259 | 0.035759 | 0.041391 | 5333 |
| 16994 | 1.01410 | 2.006 | 5.0 | 10.0 | 1 | 3 | 26.419 | 4.7 | 125.00 | 3412368 | 585824 | 17.17 | 5.026 | 1.211 | 0.164328 | 0.190208 | 111429 |
| 17053 | 0.69520 | 2.596 | 3.9 | 10.0 | 1 | 3 | 26.907 | 4.6 | 125.00 | 1642020 | 58196 | 3.54 | 3.884 | 1.189 | 0.216588 | 0.250699 | 14590 |
| 17918 | 0.25050 | 2.246 | 4.5 | 10.0 | 1 | 3 | 27.248 | 4.6 | 125.00 | 2143457 | 266206 | 12.42 | 4.489 | 1.174 | 0.189762 | 0.219648 | 58472 |
| 18439 | 1.83360 | 3.846 | 2.6 | 10.0 | 1 | 3 | 26.813 | 4.7 | 125.00 | 2770693 | 461627 | 16.66 | 2.622 | 1.193 | 0.319756 | 0.370115 | 170855 |
| 18859 | 3.23520 | 5.400 | 1.9 | 10.0 | 1 | 3 | 28.791 | 4.3 | 125.00 | 1852586 | 197827 | 10.68 | 1.867 | 1.111 | 0.482075 | 0.557998 | 110387 |
| 19586 | 0.14310 | 2.120 | 4.7 | 10.0 | 1 | 4 | 25.809 | 4.8 | 125.00 | 988829 | 7252 | 0.73 | 4.756 | 1.239 | 0.169657 | 0.196376 | 1424 |
| 20148 | 0.06340 | 0.658 | 15.2 | 10.0 | 1 | 4 | 25.852 | 4.8 | 125.00 | 881829 | 30669 | 3.48 | 15.324 | 1.237 | 0.052745 | 0.061052 | 1872 |
| 20264 | 0.11530 | 0.826 | 12.1 | 10.0 | 1 | 4 | 28.638 | 4.4 | 125.00 | 1970995 | 568551 | 28.85 | 12.207 | 1.117 | 0.073348 | 0.084899 | 48270 |
| 20278 | 2.84390 | 2.187 | 4.6 | 10.0 | 1 | 4 | 30.652 | 4.1 | 125.00 | 7122224 | 1086336 | 15.25 | 4.610 | 1.043 | 0.207860 | 0.240597 | 261369 |
| 20407 | 0.13730 | 1.088 | 9.2 | 10.0 | 1 | 4 | 27.520 | 4.5 | 125.00 | 1032889 | 107352 | 10.39 | 9.267 | 1.162 | 0.092841 | 0.107463 | 11536 |
| 21130 | 0.26910 | 0.546 | 18.3 | 10.0 | 1 | 4 | 2.427 | 30.0 | 72.81 | 331112 | 10290 | 3.11 | 18.467 | 7.676 | 0.007054 | 0.008165 | 84 |
| 21167 | 0.19870 | 0.481 | 20.8 | 10.0 | 1 | 5 | 25.573 | 4.9 | 125.00 | 2198374 | 35539 | 1.62 | 20.963 | 1.251 | 0.038141 | 0.044148 | 1569 |
| 21397 | 1.22520 | 1.836 | 5.4 | 10.0 | 1 | 5 | 24.624 | 5.1 | 125.00 | 4151243 | 347274 | 8.37 | 5.492 | 1.299 | 0.140183 | 0.162261 | 56349 |
| 21548 | 0.05012 | 0.141 | 35.0 | 4.9 | 1 | 4 | 25.042 | 5.0 | 125.00 | 1309328 | 86608 | 6.61 | 35.291 | 1.277 | 0.022185 | 0.025679 | 2224 |
| 21696 | 0.13120 | 0.709 | 14.1 | 10.0 | 1 | 5 | 27.001 | 4.6 | 125.00 | 848402 | 35589 | 4.19 | 14.221 | 1.185 | 0.059359 | 0.068708 | 2445 |
| 27187 | 1.44790 | 4.993 | 2.0 | 10.0 | 1 | 5 | 27.111 | 4.6 | 125.00 | 252743 | 4176 | 1.65 | 2.019 | 1.180 | 0.419731 | 0.485836 | 2029 |
| 27446 | 0.13490 | 0.753 | 13.3 | 10.0 | 1 | 5 | 26.515 | 4.7 | 125.00 | 1913131 | 101714 | 5.32 | 13.390 | 1.206 | 0.061909 | 0.071659 | 7289 |
| 27641 | 0.22540 | 0.514 | 19.5 | 10.0 | 1 | 5 | 27.981 | 4.5 | 125.00 | 1491592 | 57903 | 3.88 | 19.617 | 1.143 | 0.044595 | 0.051619 | 2989 |
| 27984 | 0.48350 | 0.786 | 12.7 | 10.0 | 1 | 6 | 28.817 | 4.3 | 125.00 | 3503065 | 383062 | 10.94 | 12.828 | 1.110 | 0.070232 | 0.081293 | 31140 |
| 28038 | 0.39190 | 0.610 | 16.4 | 10.0 | 1 | 6 | 29.273 | 4.3 | 125.00 | 2135445 | 293493 | 13.74 | 16.530 | 1.093 | 0.055368 | 0.064088 | 18809 |
| 29117 | 0.54580 | 0.787 | 12.7 | 10.0 | 1 | 6 | 28.542 | 4.4 | 125.00 | 3348863 | 213789 | 6.38 | 12.812 | 1.121 | 0.069650 | 0.080620 | 17236 |
| 29401 | 0.32920 | 1.724 | 5.8 | 10.0 | 1 | 6 | 28.616 | 4.4 | 125.00 | 1040738 | 19981 | 1.92 | 5.849 | 1.118 | 0.152971 | 0.177063 | 3538 |
| 29593 | 3.31700 | 10.083 | 1.0 | 10.0 | 1 | 6 | 27.633 | 4.5 | 125.00 | 3203248 | 307745 | 9.61 | 1.000 | 1.157 | 0.863936 | 1.000000 | 307745 |
| 29745 | 0.13710 | 0.458 | 15.0 | 6.9 | 1 | 6 | 28.876 | 4.3 | 125.00 | 1515984 | 102311 | 6.75 | 15.125 | 1.108 | 0.059691 | 0.069092 | 7069 |
| 30113 | 0.11730 | 1.010 | 9.9 | 10.0 | 1 | 6 | 28.135 | 4.4 | 125.00 | 809954 | 97668 | 12.06 | 9.983 | 1.137 | 0.088111 | 0.101988 | 9961 |
| 30148 | 0.12330 | 0.460 | 15.0 | 6.9 | 1 | 6 | 27.554 | 4.5 | 125.00 | 1122533 | 163604 | 14.57 | 15.125 | 1.161 | 0.056958 | 0.065929 | 10786 |
| 30256 | 0.26730 | 1.409 | 7.1 | 10.0 | 1 | 7 | 29.380 | 4.3 | 125.00 | 1343660 | 105332 | 7.84 | 7.156 | 1.089 | 0.128359 | 0.148575 | 15650 |
| 30491 | 0.18350 | 0.500 | 15.0 | 7.5 | 1 | 7 | 29.326 | 4.3 | 125.00 | 1623777 | 76762 | 4.73 | 15.125 | 1.091 | 0.060621 | 0.070169 | 5386 |
| 30972 | 0.40930 | 0.948 | 10.5 | 10.0 | 1 | 7 | 30.032 | 4.2 | 125.00 | 1865295 | 183783 | 9.85 | 10.636 | 1.065 | 0.088279 | 0.102182 | 18779 |
| 31465 | 1.35110 | 2.020 | 5.0 | 10.0 | 1 | 7 | 30.111 | 4.2 | 125.00 | 3436442 | 554189 | 16.13 | 4.992 | 1.062 | 0.188599 | 0.218303 | 120981 |
| 31516 | 0.30440 | 1.411 | 7.1 | 10.0 | 1 | 7 | 25.902 | 4.8 | 125.00 | 697244 | 43299 | 6.21 | 7.146 | 1.235 | 0.113325 | 0.131172 | 5680 |
| 31563 | 1.03370 | 2.374 | 4.2 | 10.0 | 1 | 7 | 28.635 | 4.4 | 125.00 | 1976085 | 533511 | 27.00 | 4.247 | 1.117 | 0.210786 | 0.243983 | 130168 |
| 31637 | 0.13169 | 0.723 | 13.8 | 10.0 | 1 | 5 | 26.792 | 4.7 | 125.00 | 635086 | 64002 | 10.08 | 13.946 | 1.194 | 0.060063 | 0.069523 | 4450 |
| 32372 | 0.23130 | 0.662 | 15.1 | 10.0 | 1 | 7 | 29.217 | 4.3 | 125.00 | 1111693 | 71913 | 6.47 | 15.231 | 1.095 | 0.059973 | 0.069419 | 4992 |
| 32530 | 0.67960 | 0.701 | 14.3 | 10.0 | 1 | 8 | 28.728 | 4.4 | 125.00 | 3690123 | 365409 | 9.90 | 14.384 | 1.113 | 0.062444 | 0.072278 | 26411 |
| 33076 | 0.38920 | 1.590 | 6.3 | 10.0 | 1 | 8 | 31.985 | 3.9 | 125.00 | 1182460 | 101851 | 8.61 | 6.342 | 1.000 | 0.157691 | 0.182526 | 18590 |
| 33579 | 0.55970 | 0.940 | 10.6 | 10.0 | 1 | 8 | 27.570 | 4.5 | 125.00 | 2095619 | 109425 | 5.22 | 10.727 | 1.160 | 0.080358 | 0.093014 | 10178 |
| 35287 | 0.49370 | 1.895 | 5.3 | 10.0 | 1 | 8 | 29.960 | 4.2 | 125.00 | 1045321 | 142384 | 13.62 | 5.321 | 1.068 | 0.176041 | 0.203767 | 29013 |
| 35537 | 0.23773 | 0.598 | 16.7 | 10.0 | 1 | 7 | 28.544 | 4.4 | 125.00 | 2390385 | 144850 | 6.06 | 16.861 | 1.121 | 0.052927 | 0.061263 | 8874 |
| 36022 | 0.29100 | 0.673 | 14.9 | 10.0 | 1 | 8 | 28.913 | 4.3 | 125.00 | 2606759 | 591185 | 22.68 | 14.982 | 1.106 | 0.060335 | 0.069838 | 41287 |
| 36166 | 0.84810 | 2.249 | 4.4 | 10.0 | 1 | 8 | 28.668 | 4.4 | 125.00 | 2918594 | 346068 | 11.86 | 4.483 | 1.116 | 0.199917 | 0.231403 | 80081 |
| 36424 | 0.30040 | 0.570 | 17.5 | 10.0 | 1 | 8 | 25.563 | 4.9 | 125.00 | 2203059 | 133585 | 6.06 | 17.689 | 1.251 | 0.045180 | 0.052296 | 6986 |
| 36503 | 0.06950 | 1.086 | 9.2 | 10.0 | 1 | 8 | 28.402 | 4.4 | 125.00 | 512901 | 61810 | 12.05 | 9.285 | 1.126 | 0.095641 | 0.110703 | 6843 |
| 36845 | 0.41040 | 1.131 | 8.8 | 10.0 | 1 | 9 | 28.929 | 4.3 | 125.00 | 3027707 | 244492 | 8.08 | 8.915 | 1.106 | 0.101452 | 0.117430 | 28711 |
| 37083 | 0.32840 | 2.640 | 3.8 | 10.0 | 1 | 9 | 30.261 | 4.1 | 125.00 | 1259906 | 119261 | 9.47 | 3.819 | 1.057 | 0.247714 | 0.286728 | 34195 |
| 37250 | 0.70030 | 2.216 | 4.5 | 10.0 | 1 | 9 | 28.140 | 4.4 | 125.00 | 1767905 | 99943 | 5.65 | 4.550 | 1.137 | 0.193356 | 0.223808 | 22368 |
| 38249 | 0.06339 | 0.746 | 13.4 | 10.0 | 1 | 3 | 29.767 | 4.2 | 125.00 | 836238 | 31501 | 3.77 | 13.516 | 1.075 | 0.068855 | 0.079700 | 2511 |
| 38407 | 1.47300 | 2.491 | 4.0 | 10.0 | 1 | 9 | 28.654 | 4.4 | 125.00 | 6666248 | 1893072 | 28.40 | 4.048 | 1.116 | 0.221321 | 0.256178 | 484963 |
| 38776 | 2.57950 | 3.075 | 3.3 | 10.0 | 1 | 9 | 28.612 | 4.4 | 125.00 | 3882333 | 348195 | 8.97 | 3.279 | 1.118 | 0.272808 | 0.315773 | 109951 |
| veldbl16 | 0.00055 | 0.020 | 25.0 | 0.5 | 1 | 9 | 0.360 | 30.0 | 10.80 | 464824 | 2288 | 0.49 | 25.208 | 7.676 | 0.005168 | 0.005982 | 14 |
| veldbl3 | 0.00066 | 0.033 | 25.0 | 0.8 | 1 | 9 | 2.572 | 30.0 | 77.16 | 160909 | 636 | 0.40 | 25.208 | 7.676 | 0.005168 | 0.005982 | 4 |
| veldbl5 | 0.00138 | 0.020 | 25.0 | 0.5 | 1 | 9 | 0.311 | 30.0 | 9.33 | 123180 | 1250 | 1.01 | 25.208 | 7.676 | 0.005168 | 0.005982 | 7 |

**ID**: Identification or unique identifier for each data entry; **16S qPCR (ng/ml):** DNA concentration measured using quantitative polymerase chain reaction (qPCR) in nanograms per milliliter; DNA clariostar (ng/µl): DNA concentration measured using the CLARIOstar reader in nanograms per microliter; **DNA clariostar (µl):** Volume of DNA used in microliters; **DNA clariostar (ng):** Total DNA quantity calculated based on volume and concentration; **Lib cycle:** Library cycle or sequencing cycle; **Enrich pool #:** Enrichment pool number; **Lib conc (ng/µl):** Library concentration in nanograms per microliter; **Lib pool (µl):** Volume of library pool in microliters; **Final lib pool (ng):** Total quantity of the final library pool in nanograms; **Total read pairs:** Total number of paired reads generated during sequencing; **Resfinder read pairs:** Number of read pairs that align with or are relevant to the ResFinder database (likely indicating antibiotic resistance genes); **On-target %** (Resfinder read pairs/Total read pairs * 100): Percentage of read pairs that align with the Resfinder database. **Template correction** (DNA clariostar (µl)/min(DNA clariostar (µl)): A correction factor related to the template DNA. **Lib correction** (Lib pool (µl)/min(Lib pool (µl)): A correction factor related to the library pool. **Total correction** (1/Template correction * Lib correction): Total correction factor calculated as a combination of template and library corrections. **Total Correction Maxed** (Total correction/max (Total correction)): A normalised total correction factor. **Relative Correction read pair counts** (Resfinder read pairs * Total Correction Maxed): Calculation involving the total correction factor and the number of Resfinder read pairs.

**Text S2 Methods – Data analysis**

***Resistome visualisation***

Heatmaps were used to visualise OP resistomes, using Bray-Curtis (BC) dissimilarities for hierarchical clustering between samples. Heatmaps were constructed on both the ARG level (90% identity cluster level) and the AMR phenotypic drug class level (as defined by the ResFinder database). Heatmaps were generated using the *pheatmap* package (version 1.0.12). The core resistome was defined as the set of ARGs with positive counts in at least 90% of samples in each specified group of individuals of interest with an abundance of ≥ 1.4781e^-06^ (the minimum detected ARG abundance measured in FPKM). It was visualised binarily in a Venn diagram.

***Resistome compositional analysis***

Compositional differences between samples were assessed using the BC beta diversity index which quantifies compositional dissimilarities between samples. BC dissimilarities between all samples were calculated using the *vegdist* function from the *vegan* package (version 2.5-7) ^13^. These dissimilarities in resistome composition were then visualised (FPKM at the 90% identity ARG level) using principal coordinates analysis (PCoA). Ellipses representing the 95% confidence intervals (CIs) of the centroids’ standard error were presented on the PCoA plots. All continuous livestock exposure variables were categorised into quantiles: quartiles (Q) or halves (H), where quartiles were not possible due to a high abundance of zero values. All levels were compared (i.e. Q1, Q2, Q3 and Q4 for variables split into quartiles, and H1 vs H2 for those split into halves). Statistical differences in composition between COPD case and control groups and different exposure groups (adjusted for COPD status as a covariate) were assessed using Permutational Multivariate Analysis of Variance (PERMANOVA) on the BC dissimilarities with 9999 permutations using the *adonis2* function from the *vegan* package.^14^ Homogeneity of group variances was verified using the *betadisper* function in the *vegan* package. A *p* value < 0.05 was considered statistically significant for both tests.

***Resistome differential abundance analysis***

DA analysis was subsequently employed to assess whether there were any ARGs exhibiting differential abundance between COPD cases versus controls, as well as among participants with varying degrees of exposure to livestock farms. ARG count data was abundance-prevalence filtered, maintaining ARGs that accounted for at least 0.1% of the counts in at least 15% of all samples, resulting in 30 ARGs to compare. This threshold was selected in line with filtering protocols in previous literature and after an exploration of the prevalence distribution plot (which allowed us visualise the dynamics of the ARG count population).^4^ DA analysis was performed at the ARG level, employing two distinct DA algorithms due to the substantial variability in results observed across different methodologies, as highlighted in a prior study.^15^ We implemented DESeq (from the DESeq2 R package (version 1.34.0)) and ANOVA-Like Differential Expression (ALDEx) (from the ALDEx2 R package (version 1.26.0)). DESeq estimates logarithmic (to base 2) (log2) fold changes of ARGs between groups with the assumption that the read counts follow a negative binomial distribution.^16^ In contrast, ALDEx assumes a Dirichlet distribution for each sample. The ALDEx method has been shown to produce the most consistent results across different studies in a multi-dataset review of DA analysis techniques, but is a more conservative tool than DESeq.^15^ Both employed the Benjamini-Hochberg (BH) correction to reduce the false discovery rate (FDR) and we used a significance threshold of 0.05. Livestock exposure variables treated as continuous variables in DA analyses. This approach, avoiding categorisation, preserved statistical power and averted unrealistic assumptions of homogeneity within artificially created categories. Furthermore, we incorporated COPD case-control status into the design formula of both DESeq and ALDEx analyses, establishing a multifactor design for a comprehensive examination.

The proxies selected to represent livestock exposure and used in the DA analyses were as follows: the number of distance weighted farms within 3000m, the number of horses weighted to distance in a 3000m buffer (Σ(N/m)) and the number of goats weighted to distance in a 1000m buffer (Σ(N/m)). The first variable was selected a priori as it was thought to be an accurate representation of general livestock exposure. The two latter variables were selected as they yielded *p* values < 0.2 in PERMANOVA analyses (0.077 and 0.191, respectively), indicating possible impacts on overall resistome composition.

***Resistome alpha diversity***

Within-sample diversity of ARGs was estimated by calculating the following alpha diversity indices using resistome data that had been relative rarefied, corrected for gene length and adjusted for 16S rRNA qPCR values: Shannon diversity, Simpson’s Evenness and Observed Richness. The *microbiome* package (version 1.16.0-0) was used to calculate all alpha diversity indices for each sample after transforming ARG counts to integers by multiplying by 10^9^. Alpha diversity indices for COPD cases and controls were visualised in boxplots. Statistical differences in alpha diversity metrics between COPD cases and controls were computed using a *t* test or Wilcoxon rank sum test, depending on the normality of the distribution of the values of the diversity index across the two groups.

To determine the possible relationship between livestock exposure and resistome alpha diversity we used linear regression models for the continuous livestock exposure variables (corrected for COPD status). Results were considered statistically significant at a *p* value < 0.05. Modelled concentrations of exposure to livestock-associated emissions were investigated as explanatory variables (continuous variables) in univariable analyses with alpha diversity indices as outcomes. Additionally, we investigated a livestock proxy variable in univariable analyses, specifically assessing the number of distance weighted farms within 3000m. We deemed this variable to be the most accurate representation of general livestock farm exposure, as it has been previously demonstrated to be a significant predictor of livestock-related microbial emissions.^11^

Multivariable models were applied to examine the potential impacts of exposure to specific animal species on alpha diversity, while allowing for simultaneous adjustment for COPD status and other animal species. Six animal species-specific variables were included: the number of pigs, poultry, cows, horses, goats and sheep within a 3000m radius. This enabled us to examine the potential impacts of exposure to specific animal species while correcting for the influence of other animal species and COPD status. Due to the independent nature of these variables, we were able to run these models without deviating from the model assumptions.

***Resistome and microbiome associations***

We obtained the study participants’ OP microbiota composition using 16S rRNA-based amplicon sequencing from a previous study.^4^ We refined the dataset by retaining bacterial taxa with an abundance exceeding 0.1% in at least two samples. Subsequently, we applied Hellinger transformation and agglomerated at the bacterial genus level. Resistome data was corrected as before (for input volumes (relative rarefied), gene length, and 16S qPCR bacterial levels) and clustered at the 90% identity level. For these analyses, the microbiome read count for one sample from a COPD case (sample ID: 19586) fell below the useable limit. Procrustes analysis with the *vegan* package was used to investigate potential associations between ordinations of paired microbiome and resistome samples, following the approach described in a previous study ^5^. Briefly, microbiome and resistome PCoAs were computed on the BC dissimilarities and superimposed in a symmetric Procrustes analysis using the *procrustes* function. To test for significance of fit of the Procrustes correlation, the *protest* function from the *vegan* package (version 2.5-7) with 9999 permutations was utilised, where a *p* value < 0.05 was considered statistically significant. The derived correlation matrix was used to determine whether there was an association between OP resistome and bacteriome compositions in the same individuals.

In order to elucidate the interactions between ARGs and microbiota in the OP, we conducted network analysis. We constructed a co-occurrence network showing the correlations between bacteria and ARGs, and ARGs alone. Spearman’s rank correlation coefficients between all possible pairwise combinations of ARGs and microbiota were calculated in R and correlation matrices were constructed from these (excluding self-self and entire zero-based correlations). Spearman’s rank correlation coefficients were considered robust when |ρ|> 0.6 and *p* < 0.01 (BH-corrected). Undirected networks of the significant correlations were constructed in R from the correlation matrix using the *igraph* package using the Yifan Hu layout. Networks were subsequently visualised using the interactive platform Gephi 0.9.2 (<https://gephi.org/>) with the Fruchterman-Reingold algorithm.

**Table S2.** Descriptive statistics of livestock exposure proxies computed for the study participant residential addresses (N = 69). Values are rounded to 3 decimal places.

| Livestock exposure proxies | Mean | SD | Median | 25th percentile | 75^th^ percentile | Minimum | Maximum |
| --- | --- | --- | --- | --- | --- | --- | --- |
| Distance to nearest farm (any) (-1*m) ^±^ | -437.512 | 271.636 | -387.671 | -569.085 | -248.395 | -1364.686 | -75.107 |
| Distance to nearest farm (any)(m^-1^) ^±^ | 0.003 | 0.003 | 0.003 | 0.002 | 0.004 | 0.001 | 0.013 |
| Number of farms (all) in a 3000m buffer | 86.319 | 26.928 | 86.000 | 71.000 | 106.000 | 13.000 | 137.000 |
| Number of farms (all) in a 1000m buffer | 9.000 | 6.257 | 9.000 | 4.000 | 13.000 | 0.000 | 23.000 |
| Number of farms (all) in a 500m buffer | 2.058 | 2.294 | 1.000 | 0.000 | 3.000 | 0.000 | 9.000 |
| Number of farms (all) in a 250m buffer | 0.478 | 0.994 | 0.000 | 0.000 | 1.000 | 0.000 | 5.000 |
| Number of pigs weighted to distance in a 1000m buffer (Σ(N/m)) | 10.092 | 14.325 | 3.890 | 0.000 | 13.402 | 0.000 | 63.573 |
| Number of poultry weighted to distance in a 1000m buffer (Σ(N/m)) | 54.100 | 86.205 | 2.409 | 0.000 | 72.225 | 0.000 | 382.790 |
| Number of cows weighted to distance in a 1000m buffer (Σ(N/m)) | 1.328 | 1.674 | 0.730 | 0.328 | 1.689 | 0.000 | 8.507 |
| Number of horses weighted to distance in a 1000m buffer (Σ(N/m)) | 0.124 | 0.301 | 0.035 | 0.003 | 0.150 | 0.000 | 1.887 |
| Number of goats weighted to distance in a 1000m buffer (Σ(N/m)) | 0.251 | 1.249 | 0.000 | 0.000 | 0.000 | 0.000 | 9.130 |
| Number of sheep weighted to distance in a 1000m buffer (Σ(N/m)) | 0.074 | 0.111 | 0.011 | 0.000 | 0.108 | 0.000 | 0.355 |
| Number of fur animals weighted to distance in a 1000m buffer (Σ(N/m)) | 1.224 | 5.471 | 0.000 | 0.000 | 0.000 | 0.000 | 43.006 |
| Number of pigs weighted to distance in a 3000m buffer (Σ(N/m)) | 44.159 | 28.923 | 40.168 | 22.103 | 59.913 | 0.081 | 123.806 |
| Number of poultry weighted to distance in a 3000m buffer (Σ(N/m)) | 277.434 | 256.777 | 178.424 | 111.658 | 375.765 | 0.012 | 965.464 |
| Number of cows weighted to distance in a 3000m buffer (Σ(N/m)) | 5.279 | 2.723 | 4.707 | 3.428 | 6.891 | 1.288 | 13.609 |
| Number of horses weighted to distance in a 3000m buffer (Σ(N/m)) | 0.343 | 0.346 | 0.278 | 0.181 | 0.439 | 0.007 | 2.217 |
| Number of goats weighted to distance in a 3000m buffer (Σ(N/m)) | 1.034 | 1.845 | 0.167 | 0.006 | 1.583 | 0.000 | 10.218 |
| Number of sheep weighted to distance in a 3000m buffer (Σ(N/m)) | 0.293 | 0.197 | 0.225 | 0.133 | 0.400 | 0.028 | 0.894 |
| Number of fur animals weighted to distance in a 3000m buffer (Σ(N/m)) | 4.273 | 8.593 | 1.062 | 0.006 | 4.568 | 0.000 | 52.447 |
| Number of farms (all) weighted to distance in a 1000m buffer (Σ(N/m)) | 0.016 | 0.013 | 0.012 | 0.007 | 0.023 | 0.000 | 0.054 |
| Number of farms (all) weighted to distance in a 3000m buffer (Σ(N/m)) | 0.055 | 0.020 | 0.050 | 0.040 | 0.073 | 0.012 | 0.104 |
| Dispersion-modelled endotoxin (endotoxin units (EU)/m^3^) | 0.250 | 0.137 | 0.245 | 0.161 | 0.312 | 0.031 | 0.836 |
| Dispersion-modelled PM_10_ (µg/m^3^) | 0.311 | 0.169 | 0.289 | 0.186 | 0.376 | 0.036 | 0.766 |
| LUR-modelled *E. coli* (copies/m^3^) | 3.411 | 0.447 | 3.371 | 3.149 | 3.698 | 2.577 | 4.718 |
| LUR-modelled *Staphylococcus* spp. (copies/m^3^) | 7.623 | 0.935 | 7.630 | 7.058 | 8.068 | 5.926 | 11.388 |
| LUR-modelled *tetW* (copies/m^3^) | 9.718 | 0.460 | 9.729 | 9.436 | 9.972 | 8.797 | 10.972 |
| LUR-modelled *mecA* (copies/m^3^) | 2.450 | 0.556 | 2.426 | 2.155 | 2.797 | 1.348 | 3.769 |
| RF-modelled *E. coli* (copies/m^3^) | 3.858 | 0.160 | 3.871 | 3.732 | 3.974 | 3.507 | 4.157 |
| RF-modelled  *Staphylococcus* spp. (copies/m^3^) | 7.511 | 0.173 | 7.534 | 7.379 | 7.640 | 7.003 | 7.790 |
| RF-modelled *tetW* (copies/m^3^) | 10.002 | 0.164 | 10.036 | 9.901 | 10.129 | 9.605 | 10.251 |
| RF-modelled *mecA* (copies/m^3^) | 2.784 | 0.132 | 2.791 | 2.679 | 2.881 | 2.430 | 2.992 |
| ^±^ Distance to the nearest farm values were multiplied by -1 and inversed (1/distance) so that closer farm distances are interpreted as ‘higher’ exposure values than those further away | | | | | | | |


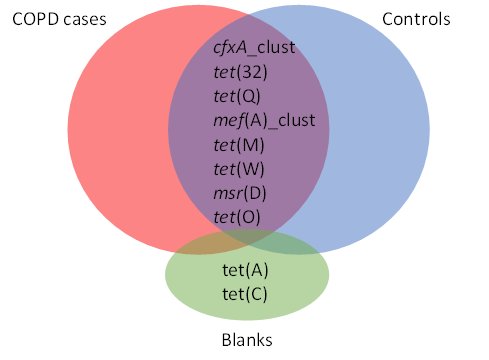
Figure S1. Venn diagram to visualise the ARG members of the core resistome of COPD cases, controls and blanks. The core resistome of COPD, control and blank groups was defined as the set of ARGs found consistently (within 90% of samples) in each group with an abundance of ≥ 1.4781e^-06^.

**
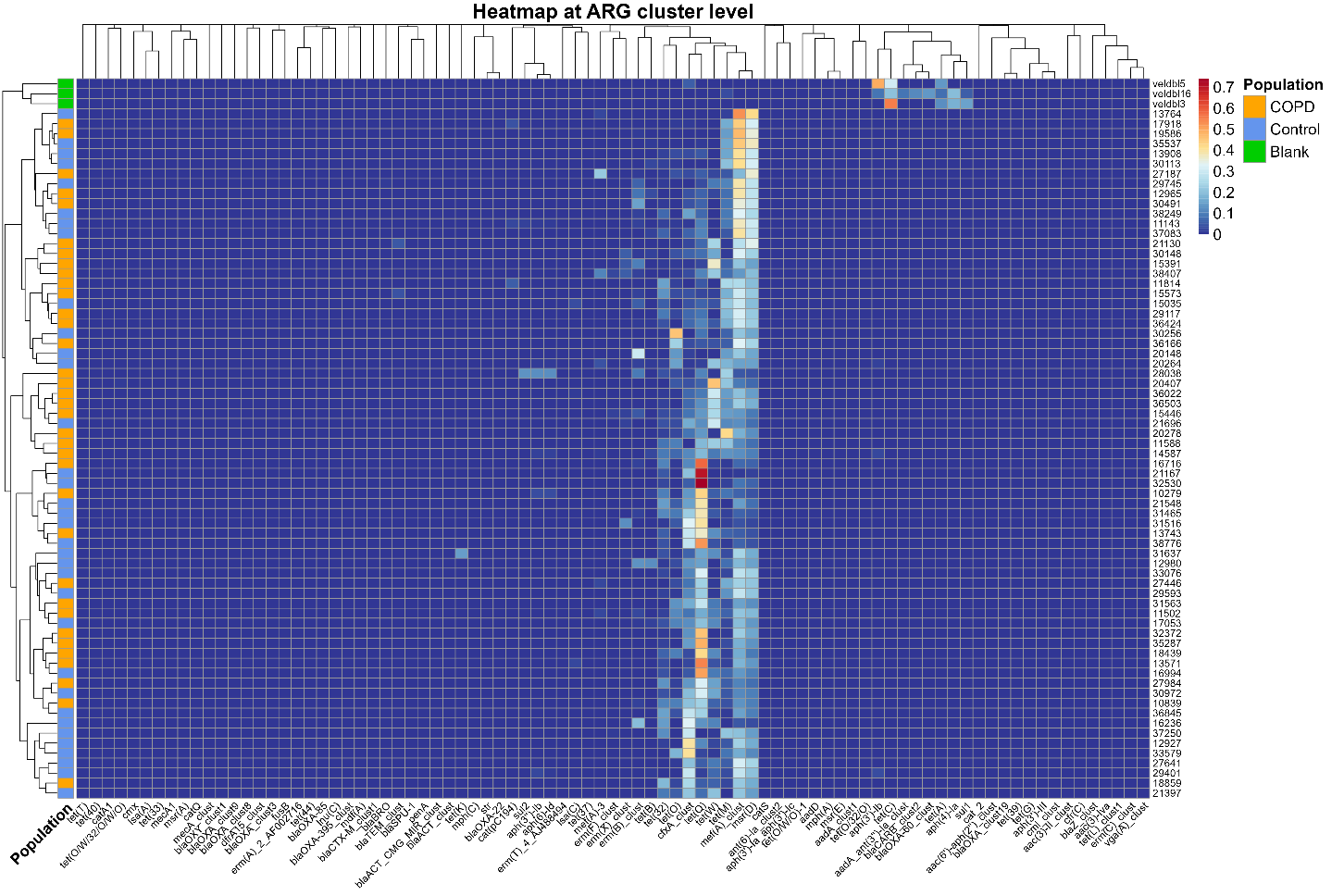
Figure S2.** Heatmap showing the abundance of ARGs identified across all samples (ARGs clustered at their 90% identity level). Raw ARG counts have been transformed to proportions of total ARGs in each sample. Colours indicate relative abundance of each ARG (columns) in each sample (rows), with red indicating high relative abundance and blue low relative abundance. Hierarchical clustering of the samples was performed based on the Bray-Curtis dissimilarity matrix.

**Table S3.** DESeq differential abundance analysis output table showing log2-fold changes of ARG clusters (90% identity level) between COPD cases and controls. Corresponding average counts of each ARG, standard errors of the log2-fold change, Wald test statistic, p value of test statistic and adjusted p value.

|  | baseMean | log2FoldChange | lfcSE | stat | pvalue | padj |
| --- | --- | --- | --- | --- | --- | --- |
| aph(3'')-Ib | 0.17039883 | -0.117861 | 2.3726886 | -0.049674 | 0.96038216 | 0.99475623 |
| aph(3')-Ia_aph(3')-Ic | 0.05573859 | -0.2009132 | 2.92675539 | -0.0686471 | 0.94527054 | 0.99475623 |
| aph(4)-Ia | 0 | NA | NA | NA | NA | NA |
| aph(6)-Id | 0.16097229 | -0.0240338 | 2.38775428 | -0.0100655 | 0.99196907 | 0.99475623 |
| blaOXA-60_clust | 0 | NA | NA | NA | NA | NA |
| blaSPU-1 | 0 | NA | NA | NA | NA | NA |
| blaTEM_clust | 0.11186023 | 1.00448205 | 2.9251751 | 0.34339211 | 0.7313035 | 0.99475623 |
| cfxA_clust | 19.2171101 | -1.2704671 | 0.49569619 | -2.5629955 | 0.01037734 | 0.11933941 |
| penA | 0 | NA | NA | NA | NA | NA |
| lsa(C) | 0.04824468 | -0.0192354 | 2.92681162 | -0.0065721 | 0.99475623 | 0.99475623 |
| erm(B)_clust | 1.58351648 | -0.9862982 | 0.96611242 | -1.0208938 | 0.30730476 | 0.99475623 |
| erm(F)_clust | 0.05914272 | 0.15810495 | 2.92681261 | 0.0540195 | 0.95691963 | 0.99475623 |
| erm(T)_4_AJ488494 | 0.05414511 | -0.1021269 | 2.92698563 | -0.0348915 | 0.97216626 | 0.99475623 |
| erm(X)_clust | 0.15954794 | 0.90523605 | 2.44831175 | 0.36973888 | 0.71157706 | 0.99475623 |
| mef(A)-3 | 0.35798125 | 1.48854583 | 1.89728024 | 0.78456824 | 0.43270675 | 0.99475623 |
| mef(A)_clust | 15.1025321 | -0.0248903 | 0.09267387 | -0.2685794 | 0.78825339 | 0.99475623 |
| msr(D) | 13.8023335 | 0.0503944 | 0.0993228 | 0.50737997 | 0.61188824 | 0.99475623 |
| cat(pC194) | 0.05338041 | 0.21190519 | 2.92657677 | 0.07240719 | 0.94227787 | 0.99475623 |
| sul1 | 0 | NA | NA | NA | NA | NA |
| sul2 | 0.00637001 | -0.1021269 | 2.92698563 | -0.0348915 | 0.97216626 | 0.99475623 |
| tet(32) | 4.91669424 | -0.019196 | 0.49982323 | -0.0384056 | 0.96936428 | 0.99475623 |
| tet(37) | 0.04901798 | -0.1921638 | 2.92692257 | -0.0656539 | 0.9476534 | 0.99475623 |
| tet(A) | 0 | NA | NA | NA | NA | NA |
| tet(B) | 0.4805488 | -0.862868 | 1.27794515 | -0.6751995 | 0.49954901 | 0.99475623 |
| tet(C) | 0 | NA | NA | NA | NA | NA |
| tet(M) | 12.5252648 | 0.95481901 | 0.32605104 | 2.92843421 | 0.00340674 | 0.078355 |
| tet(O) | 3.50868034 | -0.3348308 | 0.59479141 | -0.5629381 | 0.57347701 | 0.99475623 |
| tet(O/W/32/O/W/O) | 0.03094006 | 0.07353467 | 2.92700736 | 0.02512282 | 0.979957 | 0.99475623 |
| tet(Q) | 44.4036272 | -0.6523699 | 0.52480024 | -1.2430823 | 0.21383753 | 0.99475623 |
| tet(W) | 13.1386102 | 0.81101914 | 0.56995881 | 1.42294343 | 0.15475256 | 0.99475623 |

**baseMean:** Mean count of ARG across all samples; **log2FoldChange**: log2-transformed-fold change (L2FC) in counts for each ARG between control participants and COPD patients. A positive L2FC indicates an increase in relative abundance in the COPD group, and a negative L2FC shows a decrease; **lfcSE**: Standard errors associated with the L2FC; **stat**: Wald test statistics; **pvalue:** *p* values associated with with the Wald tests**; padj:** adjusted *p* values, corrected for multiple testing using the Benjamini-Hochberg method.

DESeq analysis requires integer count data. Post filtering (0.1% abundant, 15% prevalent) there were 30 ARG clusters remaining. However for 7 of these 30 ARG clusters, all counts were rounded to 0, hence log2 fold changes could not be computed. As a result, we have L2FC values for 23 ARG clusters.

**Table S4.** ALDEx differential abundance analysis output table showing the magnitude of the difference in abundance of ARG clusters (90% identity level) between the COPD and control groups (effect). Corresponding median clr-transformed counts of each ARG, p value of Wilcoxon rank sum test statistic and adjusted p value.

|  | rab.all | diff.btw | effect | wi.ep | wi.eBH |
| --- | --- | --- | --- | --- | --- |
| aph(3'')-Ib | -2.8237749 | -4.0650218 | -0.242936 | 0.0631121 | 0.82371251 |
| aph(3')-Ia_aph(3')-Ic | 0.94834244 | -0.4067683 | -0.0278159 | 0.82604832 | 1 |
| aph(4)-Ia | -3.6183245 | 1.11983066 | 0.05674082 | 0.74364343 | 1 |
| aph(6)-Id | -3.6694004 | -0.1203459 | -0.0083155 | 0.85727292 | 1 |
| blaOXA-60_clust | -0.5025487 | 4.12832024 | 0.19243694 | 0.27188699 | 1 |
| blaSPU-1 | -1.7533849 | -0.0856057 | -0.0040942 | 0.90025216 | 1 |
| blaTEM_clust | -3.0140437 | -0.4474179 | -0.0201335 | 0.84811673 | 1 |
| cfxA_clust | 27.518751 | -0.8597115 | -0.0792102 | 0.52152233 | 1 |
| penA | 0.02689133 | 2.24629983 | 0.12306139 | 0.38844678 | 1 |
| lsa(C) | 3.63404134 | 0.42231379 | 0.01948741 | 0.8522353 | 1 |
| erm(B)_clust | 18.9212289 | 1.55419572 | 0.07196797 | 0.57228324 | 1 |
| erm(F)_clust | -3.0244734 | -4.6261135 | -0.2169905 | 0.09976806 | 0.89693715 |
| erm(T)_4_AJ488494 | -3.8446413 | -0.8151743 | -0.0532072 | 0.74095019 | 1 |
| erm(X)_clust | -3.8344835 | 2.36336445 | 0.10651765 | 0.41756439 | 1 |
| mef(A)-3 | 13.6581904 | -1.410073 | -0.0630687 | 0.55878709 | 1 |
| mef(A)_clust | 29.4656043 | -0.539509 | -0.0592086 | 0.69769615 | 1 |
| msr(D) | 29.2477959 | -0.4274532 | -0.0505815 | 0.7103707 | 1 |
| cat(pC194) | -4.9024433 | 1.34200834 | 0.07339232 | 0.55488327 | 1 |
| sul1 | 9.45080532 | -3.2367749 | -0.1366251 | 0.39023747 | 1 |
| sul2 | -3.2387351 | -1.4344859 | -0.0857187 | 0.46826527 | 0.99210601 |
| tet(32) | 25.3106805 | -1.3303123 | -0.1147496 | 0.41900404 | 1 |
| tet(37) | 17.8353346 | -4.4919274 | -0.2014518 | 0.0864694 | 0.9273223 |
| tet(A) | -2.4998915 | -2.1852467 | -0.1174163 | 0.43522953 | 0.99955782 |
| tet(B) | 1.98425717 | -1.9639096 | -0.0767279 | 0.53511801 | 1 |
| tet(C) | -3.1986328 | 0.93575112 | 0.05992573 | 0.66364886 | 1 |
| tet(M) | 28.1717151 | 0.22039761 | 0.02836441 | 0.8915742 | 1 |
| tet(O) | 25.1970801 | -1.4849547 | -0.111693 | 0.385545 | 1 |
| tet(O/W/32/O/W/O) | -3.7874145 | 1.81051304 | 0.09755335 | 0.45315469 | 1 |
| tet(Q) | 29.3103186 | -0.2855614 | -0.0337488 | 0.84425359 | 1 |
| tet(W) | 26.7093591 | 1.43559274 | 0.1141968 | 0.38885933 | 1 |

**rab.all:** median clr value for all samples in the feature; **dif.btw:** median difference in clr values between S and NS groups; **effect:** median effect size**:** diff.btw / max(diff.win) for all instances (a negative effect value indicates an increase in relative abundance in the COPD group, and a positive is negative, the ARG is more **; wi.ep:** Expected p-value of Wilcoxon rank test; **wi.eBH:** Expected Benjamini-Hochberg corrected p-value of Wilcoxon test

**Table S5.** Tables showing the significant (BH-adjusted *p* value < 0.01) Spearman rank correlations between (**a**) bacteria-ARG and (**b**) ARG-ARG.

**a**

| **ARG** | **Bacterial genus** | **rho** | **p value** | **ARG abundance** | **Bacterial genus abundance** | **p value (BH-adj.)** |
| --- | --- | --- | --- | --- | --- | --- |
| *aac(3)-II_clust* | *Faucicola* | 0.71 | 9.60E-12 | 4.84 | 45 | 1.76E-11 |
| *blaCTX-M_clust1* | *Parascardovia* | 1.00 | 0 | 1.83 | 29 | 0 |
| *blaOXA-22* | *Streptobacillus* | 0.69 | 6.88E-11 | 11.32 | 10 | 7.57E-11 |
| *blaOXA-22* | *Alysiella* | 0.69 | 6.88E-11 | 11.32 | 19 | 7.57E-11 |
| *blaOXA-395_clust* | *Olsenella* | 0.71 | 9.60E-12 | 4.88 | 20 | 1.76E-11 |
| *cat_2* | *Streptobacillus* | 1.00 | 0 | 18.84 | 10 | 0 |
| *cat_2* | *Alysiella* | 1.00 | 0 | 18.84 | 19 | 0 |
| *catA1* | *Shuttleworthia* | 0.69 | 6.88E-11 | 1.31 | 17 | 7.57E-11 |
| *catQ* | *Cardiobacterium* | 0.70 | 2.61E-11 | 28.36 | 6 | 4.10E-11 |
| *cfr(C)* | *Faucicola* | 1.00 | 0 | 9.78 | 45 | 0 |
| *tet(37)* | *Gemella* | -0.61 | 4.43E-08 | 1858.98 | 12574 | 4.43E-08 |

**b**

| **ARG 1** | **ARG 2** | **rho** | ***p* value** | **ARG 1 abundance** | **ARG 2 abundance** | ***p* value (BH-adj.)** |
| --- | --- | --- | --- | --- | --- | --- |
| *aac(3)-II_clust* | *cfr(C)* | 0.71 | 9.60E-12 | 4.84 | 9.78 | 1.25E-11 |
| *aac(6')-aph(2'')_clust* | *aph(3')-Ib* | 1.00 | 0 | 6.77 | 1.87 | 0 |
| *aac(6')-aph(2'')_clust* | *blaOXA_clust19* | 1.00 | 0 | 6.77 | 0.96 | 0 |
| *aac(6')-aph(2'')_clust* | *catA1* | 0.71 | 9.60E-12 | 6.77 | 1.31 | 1.25E-11 |
| *aac(6')-aph(2'')_clust* | *cml_clust* | 1.00 | 0 | 6.77 | 2.42 | 0 |
| *aac(6')-aph(2'')_clust* | *tet(39)* | 1.00 | 0 | 6.77 | 1.17 | 0 |
| *aac(6')-aph(2'')_clust* | *tet(G)* | 1.00 | 0 | 6.77 | 3.29 | 0 |
| *aadA_clust1* | *blaOXY_clust1* | 0.69 | 6.88E-11 | 10.53 | 5.70 | 7.26E-11 |
| *aph(3'')-Ib* | *aph(6)-Id* | 0.66 | 1.31E-09 | 11271.35 | 10361.21 | 1.34E-09 |
| *aph(3')-Ia_aph(3')-Ic* | *aph(6)-Id* | 0.62 | 1.92E-08 | 7320.95 | 10361.21 | 1.92E-08 |
| *aph(3')-Ib* | *blaOXA_clust19* | 1.00 | 0 | 1.87 | 0.96 | 0 |
| *aph(3')-Ib* | *catA1* | 0.71 | 9.60E-12 | 1.87 | 1.31 | 1.25E-11 |
| *aph(3')-Ib* | *cml_clust* | 1.00 | 0 | 1.87 | 2.42 | 0 |
| *aph(3')-Ib* | *tet(39)* | 1.00 | 0 | 1.87 | 1.17 | 0 |
| *aph(3')-Ib* | *tet(G)* | 1.00 | 0 | 1.87 | 3.29 | 0 |
| *str* | *blaOXA-22* | 0.71 | 9.60E-12 | 8.82 | 11.32 | 1.25E-11 |
| *blaCARB_clust2* | *fusB* | 1.00 | 0 | 1.01 | 0.59 | 0 |
| *blaOXA-22* | *cat_2* | 0.69 | 6.88E-11 | 11.32 | 18.84 | 7.26E-11 |
| *blaOXA_clust19* | *catA1* | 0.71 | 9.60E-12 | 0.96 | 1.31 | 1.25E-11 |
| *blaOXA_clust19* | *cml_clust* | 1.00 | 0 | 0.96 | 2.42 | 0 |
| *blaOXA_clust19* | *tet(39)* | 1.00 | 0 | 0.96 | 1.17 | 0 |
| *blaOXA_clust19* | *tet(G)* | 1.00 | 0 | 0.96 | 3.29 | 0 |
| *mecA1* | *tet(L)_clust1* | 0.69 | 6.88E-11 | 0.48 | 23.53 | 7.26E-11 |
| *lsa(A)* | *vga(A)_clust* | 0.69 | 6.88E-11 | 2.04 | 4.90 | 7.26E-11 |
| *lsa(A)* | *cmx* | 0.69 | 6.88E-11 | 2.04 | 4.25 | 7.26E-11 |
| *lsa(A)* | *tet(33)* | 1.00 | 0 | 2.04 | 0.66 | 0 |
| *mef(A)_clust* | *msr(D)* | 0.99 | 1.40E-61 | 716727.77 | 565849.39 | 2.88E-61 |
| *mef(A)_clust* | *tet(M)* | 0.72 | 3.09E-12 | 716727.77 | 360686.47 | 5.74E-12 |
| *mph(A)* | *msr(E)* | 1.00 | 0 | 7.73 | 10.18 | 0 |
| *msr(D)* | *tet(M)* | 0.73 | 1.66E-12 | 565849.39 | 360686.47 | 3.23E-12 |
| *vga(A)_clust* | *tet(33)* | 0.69 | 6.88E-11 | 4.90 | 0.66 | 7.26E-11 |
| *vga(A)_clust* | *tet(44)* | 0.71 | 9.60E-12 | 4.90 | 4.02 | 1.25E-11 |
| *catA1* | *cml_clust* | 0.71 | 9.60E-12 | 1.31 | 2.42 | 1.25E-11 |
| *catA1* | *tet(39)* | 0.71 | 9.60E-12 | 1.31 | 1.17 | 1.25E-11 |
| *catA1* | *tet(G)* | 0.71 | 9.60E-12 | 1.31 | 3.29 | 1.25E-11 |
| *cml_clust* | *tet(39)* | 1.00 | 0 | 2.42 | 1.17 | 0 |
| *cml_clust* | *tet(G)* | 1.00 | 0 | 2.42 | 3.29 | 0 |
| *cmx* | *tet(33)* | 0.69 | 6.88E-11 | 4.25 | 0.66 | 7.26E-11 |
| *tet(39)* | *tet(G)* | 1.00 | 0 | 1.17 | 3.29 | 0 |

Table S6. Univariable PERMANOVAs to compare OP resistome composition between quantiles of all listed livestock-related characteristics and modelled livestock exposures. The assumption of homogeneity of variance (*betadisper* function) was met for all variables (*p* value > 0.05). The *p* values underwent correction for multiple testing using the Benjamini-Hochberg method (*p* value (BH-adj)).

| **Livestock exposure proxy** | **R^2^** | ***p* value** | ***p* value (BH-adj)** |
| --- | --- | --- | --- |
| Distance to nearest farm (any) (-1*m) | 0.040 | 0.575 | 0.893 |
| Distance to nearest farm (any)(m^-1^) | 0.040 | 0.575 | 0.893 |
| Number of farms (all) in a 3000m buffer | 0.042 | 0.514 | 0.893 |
| Number of farms (all) in a 1000m buffer | 0.024 | 0.972 | 0.972 |
| Number of farms (all) in a 500m buffer | 0.022 | 0.131 | 0.749 |
| Number of farms (all) in a 250m buffer | 0.022 | 0.145 | 0.749 |
| Number of pigs weighted to distance in a 1000m buffer (Σ(N/m)) | 0.009 | 0.788 | 0.915 |
| Number of poultry weighted to distance in a 1000m buffer (Σ(N/m)) | 0.007 | 0.887 | 0.918 |
| Number of cows weighted to distance in a 1000m buffer (Σ(N/m)) | 0.039 | 0.589 | 0.893 |
| Number of horses weighted to distance in a 1000m buffer (Σ(N/m)) | 0.031 | 0.850 | 0.915 |
| Number of goats weighted to distance in a 1000m buffer (Σ(N/m)) | 0.014 | 0.360 | 0.881 |
| Number of sheep weighted to distance in a 1000m buffer (Σ(N/m)) | 0.021 | 0.164 | 0.749 |
| Number of pigs weighted to distance in a 3000m buffer (Σ(N/m)) | 0.042 | 0.510 | 0.893 |
| Number of poultry weighted to distance in a 3000m buffer (Σ(N/m)) | 0.038 | 0.636 | 0.893 |
| Number of cows weighted to distance in a 3000m buffer (Σ(N/m)) | 0.037 | 0.685 | 0.893 |
| Number of horses weighted to distance in a 3000m buffer (Σ(N/m)) | 0.063 | 0.084 | 0.749 |
| Number of goats weighted to distance in a 3000m buffer (Σ(N/m)) | 0.031 | 0.854 | 0.915 |
| Number of sheep weighted to distance in a 3000m buffer (Σ(N/m)) | 0.038 | 0.664 | 0.893 |
| Number of farms (all) weighted to distance in a 1000m buffer (Σ(N/m)) | 0.032 | 0.847 | 0.915 |
| Number of farms (all) weighted to distance in a 3000m buffer (Σ(N/m)) | 0.039 | 0.611 | 0.893 |
| Dispersion-modelled endotoxin concentration (endotoxin units (EU)/m^3^) | 0.063 | 0.088 | 0.749 |
| Dispersion-modelled PM_10_ concentration (μg/m^3^) | 0.046 | 0.401 | 0.881 |
| LUR-modelled *E. coli* (ln copies/m^3^) | 0.049 | 0.293 | 0.845 |
| LUR-modelled *Staphylococcus* spp. (ln copies/m^3^) | 0.045 | 0.411 | 0.881 |
| LUR-modelled *tetW* (ln copies/m^3^) | 0.055 | 0.185 | 0.749 |
| LUR-modelled *mecA* (ln copies/m^3^) | 0.049 | 0.293 | 0.845 |
| RF-modelled *E. coli* (ln copies/m^3^) | 0.066 | 0.059 | 0.749 |
| RF-modelled *Staphylococcus* spp. (ln copies/m^3^) | 0.034 | 0.763 | 0.915 |
| RF-modelled *tetW* (ln copies/m^3^) | 0.049 | 0.310 | 0.845 |
| RF-modelled *mecA* (ln copies/m^3^) | 0.054 | 0.200 | 0.749 |

**Table S7:** Univariable linear model estimates for OP resistome alpha diversity (Shannon, Simpson’s evenness and observed richness indices), in relation to general livestock exposure and modelled microbial concentrations, corrected for COPD case control status. The *p* values underwent correction for multiple testing using the Benjamini-Hochberg method (*p* value (BH-adj)).

|  | **Shannon** | | | **Simpson Evenness** | | | **Observed** | | |
| --- | --- | --- | --- | --- | --- | --- | --- | --- | --- |
|  | **β** | ***p* value** | ***p* value (BH-adj)** | **β** | ***p* value** | ***p* value (BH-adj)** | **β** | ***p* value** | ***p* value (BH-adj)** |
| **N of distance weighted farms within 3000m** | 1.460 | 0.570 | 0.700 | 0.820 | 0.040 | 0.150 | -162.320 | 0.100 | 0.573 |
| **Dispersion-modelled endotoxin** | 0.485 | 0.209 | 0.700 | 0.029 | 0.631 | 0.684 | 13.198 | 0.376 | 0.645 |
| **Dispersion-modelled PM_10_** | 0.310 | 0.330 | 0.700 | -0.02 | 0.680 | 0.684 | 15.120 | 0.210 | 0.573 |
| **LUR-modelled *E. coli*** | 0.104 | 0.378 | 0.700 | 0.012 | 0.512 | 0.625 | 1.0150 | 0.824 | 0.824 |
| **RF-modelled *E. coli*** | -0.001 | 0.997 | 0.997 | 0.064 | 0.223 | 0.409 | -17.514 | 0.174 | 0.573 |
| **LUR-modelled *Staphylococcus* spp.** | 0.071 | 0.208 | 0.700 | 0.021 | 0.014 | 0.150 | -1.226 | 0.574 | 0.702 |
| **RF-modelled *Staphylococcus* spp.** | 0.176 | 0.566 | 0.700 | 0.082 | 0.087 | 0.239 | -12.932 | 0.273 | 0.600 |
| **LUR-modelled *tetW*** | 0.071 | 0.539 | 0.700 | 0.025 | 0.158 | 0.347 | -3.418 | 0.441 | 0.645 |
| **RF-modelled *tetW*** | 0.111 | 0.736 | 0.810 | 0.039 | 0.450 | 0.618 | -9.219 | 0.469 | 0.645 |
| **LUR-modelled *mecA*** | 0.067 | 0.483 | 0.700 | 0.016 | 0.268 | 0.422 | -1.238 | 0.736 | 0.810 |
| **RF-modelled *mecA*** | 0.233 | 0.563 | 0.700 | 0.128 | 0.040 | 0.150 | -22.941 | 0.137 | 0.573 |

**Table S8.** Multivariable linear model estimates for OP resistome alpha diversity (**a**) shannon index, (**b**) simpson evenness and (**c**) observed richness in relation to animal species-specific exposures, corrected for COPD case control status.

**a**

|  | Shannon diversity index | | |
| --- | --- | --- | --- |
| Predictors | **Estimates** | **Std. Error** | ***p* value** |
| Intercept | 2.23 | 0.177 | <0.001 |
| COPD control vs case | -0.21 | 0.110 | 0.072 |
| Number of distance weighted pigs within 3000m | 0.00 | 0.002 | 0.663 |
| Number of distance weighted poultry within 3000m | 0.00 | 0.000 | 0.592 |
| Number of distance weighted cows within 3000m | -0.01 | 0.027 | 0.637 |
| Number of distance weighted horses within 3000m | 0.12 | 0.232 | 0.613 |
| Number of distance weighted goats within 3000m | 0.03 | 0.044 | 0.506 |
| Number of distance weighted sheep within 3000m | 0.06 | 0.329 | 0.859 |

**b**

|  | Simpson Evenness diversity index | | |
| --- | --- | --- | --- |
| Predictors | **Estimates** | **Std. Error** | ***p* value** |
| Intercept | 0.134 | 0.025 | 0.000 |
| COPD control vs case | -0.023 | 0.016 | 0.144 |
| Number of distance weighted pigs within 3000m | 0.000 | 0.000 | 0.887 |
| Number of distance weighted poultry within 3000m | 0.000 | 0.000 | 0.355 |
| Number of distance weighted cows within 3000m | 0.001 | 0.004 | 0.740 |
| Number of distance weighted horses within 3000m | 0.053 | 0.033 | 0.116 |
| Number of distance weighted goats within 3000m | 0.006 | 0.006 | 0.315 |
| Number of distance weighted sheep within 3000m | 0.013 | 0.047 | 0.779 |

**c**

|  | Observed diversity index | | |
| --- | --- | --- | --- |
| Predictors | **Estimates** | **Std. Error** | ***p* value** |
| Intercept | 56.271 | 6.624 | 0.000 |
| COPD control vs case | -5.219 | 4.119 | 0.210 |
| Number of distance weighted pigs within 3000m | -0.024 | 0.087 | 0.782 |
| Number of distance weighted poultry within 3000m | 0.011 | 0.009 | 0.200 |
| Number of distance weighted cows within 3000m | -0.701 | 0.994 | 0.483 |
| Number of distance weighted horses within 3000m | -10.644 | 8.705 | 0.226 |
| Number of distance weighted goats within 3000m | 1.274 | 1.662 | 0.446 |
| Number of distance weighted sheep within 3000m | -7.919 | 12.315 | 0.523 |

**References**

1. Zankari E, Hasman H, Cosentino S *et al.* Identification of acquired antimicrobial resistance genes. *J. Antimicrob. Chemother.* 2012; **67**: 2640–2644.

2. Bushnell, B. BBMap download | SourceForge.net [WWW Document]. URL https:// sourceforge.net/projects/bbmap/. (2013).

3. Macedo G, van Veelen HPJ, Hernandez-Leal L *et al.* Targeted metagenomics reveals inferior resilience of farm soil resistome compared to soil microbiome after manure application. *Sci. Total Environ.* 2021; **770**: 145399.

4. van Kersen W, Bossers A, de Steenhuijsen Piters WAA *et al.* Air pollution from livestock farms and the oropharyngeal microbiome of COPD patients and controls. *Environ Int.* 2022; **169**: 107497.

5. Munk, P. *et al.* Abundance and diversity of the faecal resistome in slaughter pigs and broilers in nine European countries. *Nat Microbiol.* 2018; **3**: 898–908.

6. McMurdie PJ & Holmes S. phyloseq: An R Package for Reproducible Interactive Analysis and Graphics of Microbiome Census Data. *PLoS ONE* **8**, e61217 (2013).

7. de Rooij MMT, Smit LAM, Erbrink HJ *et al.* Endotoxin and particulate matter emitted by livestock farms and respiratory health effects in neighboring residents. *Environ Int.* 2019; **132**: 105009.

8. Gibbs SG, Green CF, Tarwater PM *et al.* Isolation of Antibiotic-Resistant Bacteria from the Air Plume Downwindof a Swine Confined or Concentrated Animal Feeding Operation. *Environ Health Perspect.* 2006; **114**: 1032–1037.

9. McEachran AD Blackwell BR, Hanson JD *et al.* Antibiotics, Bacteria, and Antibiotic Resistance Genes: Aerial Transport from Cattle Feed Yards via Particulate Matter. *Environ Health Perspect.* 2015; **123:** 337–343.

10. Nguyen XD, Zhao Y, Evans JD *et al*. Survival of Escherichia coli in Airborne and Settled Poultry Litter Particles. *Animals.* 2022; **12**: 284.

11. de Rooij MMT, Heederik DJJ, van Nunen EJHM *et al.* Spatial Variation of Endotoxin Concentrations Measured in Ambient PM10 in a Livestock-Dense Area: Implementation of a Land-Use Regression Approach. *Environ Health Perspect.* 2018; **126**: 017003.

12. Cornu Hewitt B, Smit LAM, van Kersen W *et al.* Residential exposure to microbial emissions from livestock farms: Implementation and evaluation of land use regression and random forest spatial models. *Environ Pollut.* 2024; **346**, 123590.

13. Oksanen J, Blanchet FG, Kindt R *et al.* vegan: Community Ecology Package. R package. (2019).

14. Anderson MJ. A new method for non-parametric multivariate analysis of variance. *Austral Ecol.* 2001; **26**: 32–46.

15. Nearing JT, Douglas GM, Hayes M *et al*. Microbiome differential abundance methods produce disturbingly different results across 38 datasets. *Nat Commun*. 2022; 13: 342.

16. Lin H, Peddada SD. Analysis of compositions of microbiomes with bias correction. *Nat Commun.* 2020; **11**: 3514.
